# High-Resolution Melting Molecular Signatures for Rapid Identification of Human Papillomavirus Genotypes

**DOI:** 10.1371/journal.pone.0042051

**Published:** 2012-08-20

**Authors:** Ta-Hsien Lee, Tzong-Shoon Wu, Ching-Ping Tseng, Jiantai Timothy Qiu

**Affiliations:** 1 Graduate Institute of Biomedical Sciences, College of Medicine, Taoyuan, Taiwan; 2 Department of Biomedical Sciences, College of Medicine, Taoyuan, Taiwan; 3 Department of Medical Biotechnology and Laboratory Science, College of Medicine, Taoyuan, Taiwan; 4 Molecular Medicine Research Center, Chang Gung University, Taoyuan, Taiwan; 5 Department of Obstetrics and Gynecology, Chang Gung Memorial Hospital, Taoyuan, Taiwan; National Institute of Health - National Cancer Institute, United States of America

## Abstract

**Background:**

Genotyping of human papillomarvirus (HPV) is crucial for patient management in a clinical setting. This study accesses the combined use of broad-range real-time PCR and high-resolution melting (HRM) analysis for rapid identification of HPV genotypes.

**Methods:**

Genomic DNA sequences of 8 high-risk genotypes (HPV16/18/39/45/52/56/58/68) were subject to bioinformatic analysis to select for appropriate PCR amplicon. Asymmetric broad-range real-time PCR in the presence of HRM dye and two unlabeled probes specific to HPV16 and 18 was employed to generate HRM molecular signatures for HPV genotyping. The method was validated via assessment of 119 clinical HPV isolates.

**Results:**

A DNA fragment within the L1 region was selected as the PCR amplicon ranging from 215–221 bp for different HPV genotypes. Each genotype displayed a distinct HRM molecular signature with minimal inter-assay variability. According to the HRM molecular signatures, HPV genotypes can be determined with one PCR within 3 h from the time of viral DNA isolation. In the validation assay, a 91% accuracy rate was achieved when the genotypes were in the database. Concomitantly, the HRM molecular signatures for additional 6 low-risk genotypes were established.

**Conclusions:**

This assay provides a novel approach for HPV genotyping in a rapid and cost-effective manner.

## Introduction

Human papillomavirus (HPV), a small and nonenveloped double-stranded DNA virus, is established as the key etiological factor in cervical neoplasms [1]–[3]. The recognition of the central role of HPV infections in the etiology of virtually all cervical cancers has dramatically changed the perspectives of diagnoses and prevention of this neoplasia [4], [5]. Currently, HPV DNA testing plays a pivotal role for atypical squamous cells of undetermined significance, primary screening in conjunction with cytology for the detection of cervical cancer and cervical intraepithelial neoplasia, and follow-up in a variety of clinical settings [6]–[12]. Genotyping assays are also instrumental in assessing the impact of HPV vaccination on the risk of acquisition and on the distribution of individual HPV types in a population [8], [9], [13], [14].

HPV infection can be monitored by detection of thirteen high-risk oncogenic HPV types (HPV16, 18, 31, 33, 35, 39, 45, 51, 52, 56, 58, 59 and 68) using a commercially available HPV testing method such as the Hybrid Capture 2 assay (Digene Corporation, Gaithersburg, MD), the only HPV assay approved by the US Food and Drug Administration [11], [15], [16]. However, information on HPV genotype is lacking in the cocktail detection method. Other detection systems that determine HPV genotype include nonamplification Southern and dot blot hybridization with type-specific probes [17], type-specific PCR [18], [19], and broad-range PCR [5], [20]. The disadvantage of type-specific PCR is that multiple hybridization reactions are needed to access multiple HPV genotypes in a single sample, while broad-range PCR such as MY09/11 has the drawback of a large PCR fragment with less sensitivity [21]. Under these circumstances, there is a clinical demand for developing a simple and accurate method for identification of infecting HPV genotype with high specificity and sensitivity.

Recently, a high-resolution melting (HRM) analysis method which incorporates double-stranded DNA saturating dye and specifically designed data collection and analyzing software has been developed. HRM analysis was first developed as a closed-tube technology for genotyping DNA variants and mutation screening with advantages over the other techniques such as high-throughput, rapid and non-destructive nature [22]–[29]. Instead of using a labeled primer to analyze the domain in which the mutation resides, Wittwer and his coworkers developed HRM analysis using a saturation dye LCGreen I to substitute the need of labeled primer [28]. The combined use of real-time PCR and HRM for analysis of microbial DNA results in distinguishable HRM profiles and generates unique molecular fingerprints that facilitates its practical applications such as quantification of pathogen load and microbial species identification [30]–[35]. If required, heteroduplex formation or multiple PCR fragments can be employed to distinguish microbial species with closely similar HRM profile [30], [31], [36]. A modified HRM protocol incorporating unlabeled probes has also been reported for genotyping of herpes simplex virus that provides an alternative to detect and genotype low copies of viral infection [23].

In this study, we reported a novel method based on the use of HRM analysis and unlabeled probes to rapidly identify and differentiate HPV genotypes in clinical specimens. Without multiplexing, HPV genotypes can be completed with one PCR within 3 h from the time of viral DNA isolation.

## Materials and Methods

### Patients

All cervical samples were collected from the Department of Obstetrics and Gynecology, Chang Gung Memorial Hospital with the approval by the Institutional Review Board (IRB 99-0112B) and with informed consent from the patients. The genomic DNAs of 140 consecutive HPV clinical specimens were obtained from the sample bank for this study. The individuals who performed the experiments do not know the genotype of these clinical specimens until completion of HRM analysis.

### Materials

The QIAamp DNA mini kit was purchased from Qiagen (Hilden, Germany). The LightCycler 480 and the LightCycler 480 High Resolution Melting Master were purchased from Roche Applied Science (Mannheim, Germany). The T&A cloning vector was purchased from RBC Bioscience (Taipei, Taiwan). The McTaq DNA polymerase was purchased from One-Star Biotechnology (Taipei, Taiwan). The UniPOL-Long Range PCR enzyme mix containing Taq DNA polymerase and AccuPOL with 3′→5′ exonuclease activity was purchased from Ampliqon ApS (Skovlunde, Denmark). The EasyChip HPV genotyping system was purchased from KingCar (Yilan, Taiwan).

### Genomic DNA isolation

The QIAamp DNA mini kit was used to extract DNA from cervical specimen and the genomic DNA was eluted in 50 µl of elution buffer. The quality of the extracted DNA was checked by conventional PCR to amplify a housekeeping gene glyceraldehyde 3-phosphate dehydrogenase (GAPDH) in a 50 µl of reaction containing 5 µl of 10× PCR buffer (20 mM MgCl_2_), 2 µl of 10 mM dNTP, 0.5 µl of McTaq DNA polymerase (5 U/µl), 2 µl of 5 µM forward primer GAPDH-F, 2 µl of 5 µM reverse primer GAPDH-R (Table 1) and 2 µl of template DNA. The PCR condition was 95°C for 5 min followed by 50 cycles of 95°C for 30 sec, 60°C for 40 sec, and 72°C for 60 sec. An additional 3 min of extension at 72°C was performed after the last PCR cycle to replenish PCR products followed by cooling at 4°C.

**Table 1:** The primers and unlabeled probes sequences.

Primer/probe type	Primer/probe name	Sequences
Forward primer	FRG5	5′-CARTTATTTAATAARCCATATTGGITACA-3′
Reverse primer	FRG2	5′-TGAAAWATAAAYTGYAAATCATATTCCTC-3′
Forward primer	GAPDH-F2	5′-CCCTGGAGCCTTCAGTTGCAGCCA-3′
Reverse primer	GAPDH-R5	5′-CGTTCTCAGCCTTGACGGTGCCAT-3′
Unlabeled probe	HPV-16-UP	5′-ATTATGTGCTGCCATATCTACTTCAGAA-3′
Unlabeled probe	HPV-18-UP	5′-TGCTTCTACACAGTCTCCTGTACCTGGGCA-3′

### EasyChip genotyping

The HPV DNA testing was done routinely for patients who attended our dysplasia unit. Clinical DNA samples were subjected to EasyChip assay platform for HPV genotyping. The details of HPV blot format and typing procedure were described previously [37]. Briefly, 20 µl of the denatured amplicon was hybridized to the blot and the genotype was determined using streptavidin-alkaline phosphatase conjugate and substrate. After the blot was dried, the HPV genotypes displayed on the blot were determined using a standard visual assessment protocol.

### Plasmid construction of L1 fragment from various HPV genotypes

Partial HPV L1 region was amplified by PCR using the clinical DNA samples of HPV16, 18, 39, 45, 52, 56, 58 and 68 and the primer pair FRG5/FRG2 (Table 1). Briefly, the PCR reaction (50 µl) was composed of 5 µl of 10× UniPOL buffer B (15 mM MgCl_2_), 1 µl of 25 mM MgCl_2_, 2 µl of 10 mM dNTP, 0.5 µl of the UniPOL-Long Range PCR Enzyme mix (5 U/µl), 2 µl of forward primer FRG5 (5 µM), 2 µl of reverse primer FRG2 (5 µM) and 2 µl of template DNA. The PCR condition was 95°C for 5 min followed by 50 cycles of 95°C for 30 sec, 46°C for 40 sec, and 72°C for 30 sec. An additional 3 min of extension at 72°C was performed after the last PCR cycle to replenish PCR product followed by cooling at 4°C. The PCR product was then cloned into the T&A cloning vector as described by the manufacturer's instruction (RBC Bioscience) and was confirmed by DNA sequencing.

### Symmetric and asymmetric broad-range real-time PCR and HRM analysis

For symmetric amplification of the HPV genomic DNA, broad-range real-time PCR was performed in a 384-well format using LightCycler 480. Briefly, the PCR reaction (20 µl) was composed of 10 µl of 2× HRM master mix, 2 µl of 25 mM MgCl_2_, 0.8 µl of 5 µM forward primer FRG5, 0.8 µl of 5 µM reverse primer FRG2, and 2 µl of template DNA. The amplification condition was optimized for the use of FastStart Taq DNA polymerase by incubating the reaction mixtures at 95°C for 15 min, followed by 50 cycles of 95°C for 15 sec, 46°C for 20 sec, and 72°C for 30 sec with the ramp to 95°C at 4.8°C/s, to 46°C at 2.5°C/s, and to 72°C at 4.8°C/s. An additional 1 min at 72°C was added to replenish the PCR product.

For asymmetric amplification of HPV genomic DNA, the PCR reaction (20 µl) was composed of 10 µl of 2× HRM master mix, 2 µl of 25 mM MgCl_2_, 0.4 µl of 2.5 µM forward primer FRG5, 2 µl of 5 µM reverse primer FRG2, 2 µl of 5 µM unlabeled probe for HPV16 and HPV18 when indicated, and 2 µl of template DNA. The unlabeled probe was modified by C6-amine or inverted dT at the 3′-end to prevent the probe from self-extension. The amplification condition was optimized for the use of FastStart Taq DNA polymerase by incubating the reaction mixtures at 95°C for 15 min, followed by 65 cycles of 95°C for 15 sec, 46°C for 20 sec, and 72°C for 30 sec with the ramp to 95°C at 4.8°C/s, to 46°C at 2.5°C/s, and to 72°C at 4.8°C/s. An additional 1 min at 72°C was added to replenish PCR product.

For HRM analysis, the PCR product was denatured by rising temperature to 95°C at 4.8°C/s and was then cool down to 55°C at 2.5°C/s for hybridization. The melting curve was acquired by increasing the temperature from 55°C to 95°C at a ramp rate of 4.8°C/s with 25 acquisitions per degree of temperature.

## Results

**Figure 1:**
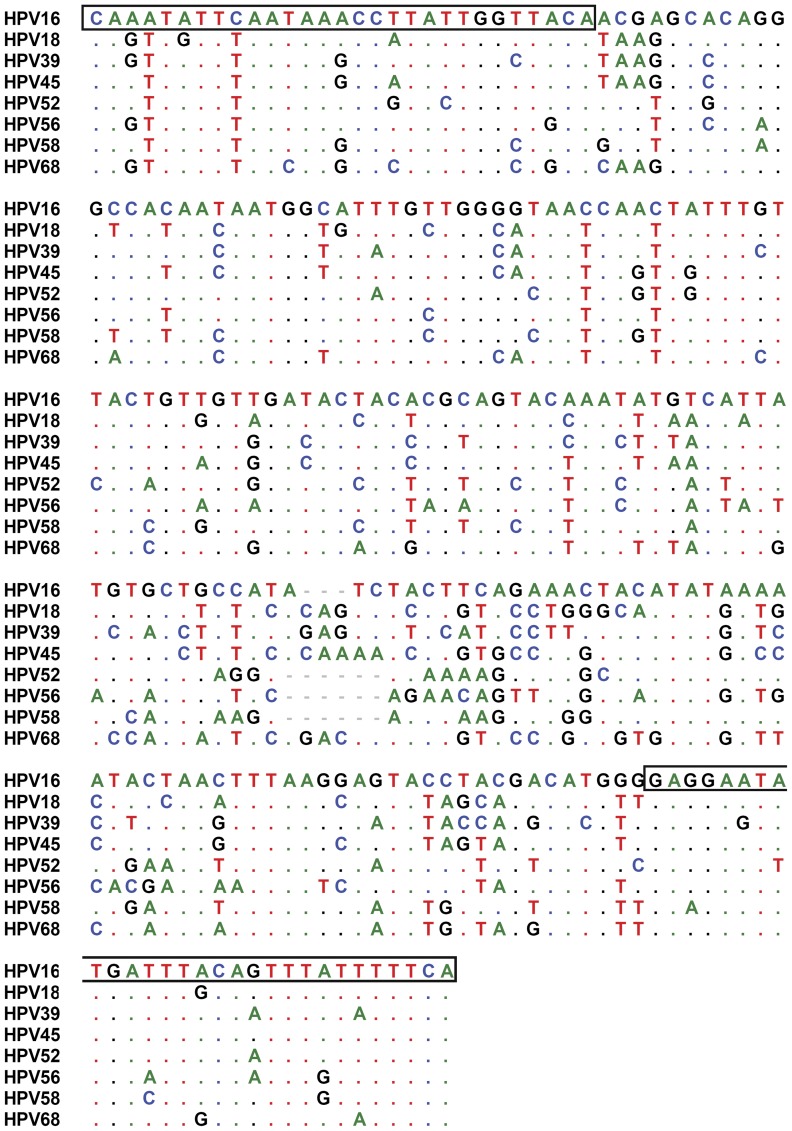
Sequence alignments of the L1 fragment PCR amplicons. Sequence alignments of the PCR amplicons corresponding to nt 6895 to 7115 of the L1 fragment (accession number NC_001526.1) for the indicated HPV genotypes. Only the sequences showing differences from the first sequence are shown. Nucleotides identical to the nucleotide in the first sequence are indicated by dots. The underlined sequences were used for the design of primers FRG5 and FRG2.

To assess the combined use of real-time PCR and HRM analysis for rapid detection and differentiation of HPV genotypes, genomic DNA sequences for 8 high-risk HPV genotypes (HPV16, 18, 39, 45, 52, 56, 58 and 68) were subject to bioinformatic analysis. These genotypes represent 98% and 75% of the clinical isolates in Southeast Asia and Europe/US, respectively [37], [38]. After multiple-sequence alignment of HPV genomic DNA sequences from different genotypes using the Vector NTI and BioEdit software packages, the fragments that are highly degenerated and flanked by conserved DNA sequences were chosen as the candidate targets for design of broad-range PCR to amplify HPV genomic DNA. Accordingly, a L1 fragment corresponding to nt 6895 to nt 7115 of HPV16 (accession number NC_001526.1) with the size ranging from 215 to 221 bp for different HPV genotypes was found to fulfill our selection criteria (Fig. 1). Due to the conserved nature of these nucleotides among various HPV genotypes, the nt 6895 to 6923 and nt 7087 to 7115 were selected to design the forward primer FRG5 and reverse primer FRG2, respectively (Table 1).

**Figure 2:**
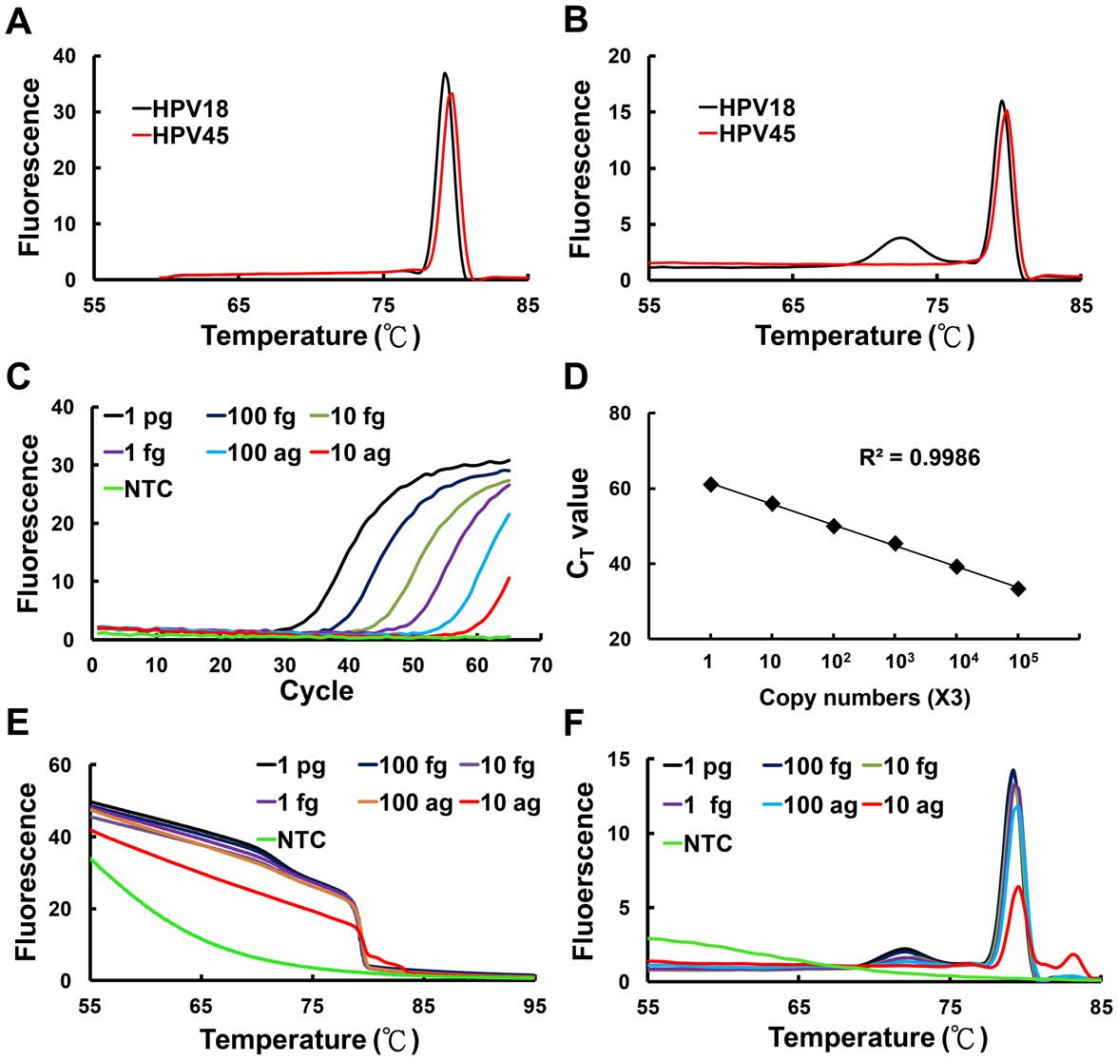
Asymmetric broad-range real-time PCR of L1 PCR amplicon. A and B. The L1 fragments of HPV18 and HPV45 were subject to symmetric (panel A) or asymmetric (panel B) PCR amplification using the primers FRG5 and FRG2. An unlabeled probe complementary to HPV18 target sequence was included in the reaction during asymmetric PCR. HRM analysis was then performed and the derivative plots for the indicated genotypes were shown. C–F. Serial dilution of the plasmid (from 1 pg to 10 ag) harboring the L1 fragment of HPV18 were used as the templates for asymmetric broad-range real-time PCR with the primer pair FRG5 and FRG2. (C) A typical LightCycler 480 amplification plot. (D) The C_t_ plotted against the plasmid DNA concentration. (E) A typical high-resolution melting plot. (F) A typical high-resolution derivative plots. NTC, no-template control.

We determined whether symmetric PCR of the L1 fragment followed by HRM analysis provides distinguishable melting profiles for HPV genotyping. To facilitate the assay, the PCR products corresponding to L1 fragment of various HPV genotypes were subcloned into the T&A cloning vector. These plasmids were used as the template for PCR amplification using the primers FRG5 and FRG2. Despite that most of the HPV genotypes displayed unique HRM profiles, the melting profiles for some HPV genotypes were not distinguishable. For example, HPV18 and HPV45 exhibited almost identical derivative plots and the genotypes were not likely to be determined accordingly (Fig. 2A).

To overcome this problem, we determined whether appropriate use of type-specific unlabeled probe(s) generates HRM profiles sufficient for differentiating HPV genotypes. To facilitate the binding of unlabeled probe to the PCR product, asymmetric PCR was performed to selectively amplify the DNA strain complementary to the unlabeled probe. An unlabeled probe specific to HPV18 was designed (Table 1) and was added into the asymmetric PCR reaction. As illustrated in Fig. 2B, the HPV18-specific unlabeled probe resulted in an additional melting peak for HPV18 and thereby the genotypes of HPV18 and HPV45 can be unambiguously differentiated (Fig. 2B).

The detection limit of this method was evaluated by asymmetric PCR amplification of 10-fold serially diluted plasmid DNA (from 1 pg to 10 ag) that contained 221-bp of HPV18 L1 fragment. The amplification reaction was performed in the presence of 40 ng of human genomic DNA to mimic the co-presence of HPV and human genomic DNA in clinical specimens. As revealed by amplification plots, as little as 10 ag plasmid DNA equivalent to 3 copies of HPV DNA was detectable in this assay condition (Fig. 2C). The standard curve showed a dynamic linear range for quantification across 6 logs of DNA concentrations and had a correlation coefficient of 0.9986 (Fig. 2D). Notably, both the melting and derivative plots were consistent when the amounts of template DNA from 1 pg to 10 ag were subject to asymmetric PCR amplification (Fig. 2E and 2F).

It is known that the last 6–7 nucleotides of PCR primers are critical for amplification specificity and efficiency [39], [40]. Due to the degenerative nature of the FRG5 and FRG2 primers and the HPV68 genomic DNA carrying 2 mismatches at the last 7 nucleotides of the FRG5 primer region, we determined whether the efficiency for amplification of HPV68 DNA is affected. When the amounts of template DNA ranging from 1 pg to 100 ag were used, PCR amplification of HPV68 DNA was as efficient as for HPV18 DNA with no potential skewing. The presence of human genomic DNA also had no effect on amplification of HPV18 and HPV68 DNA (Fig. S1). However, 10 ag of plasmid template DNA for HPV18 but not HPV68 could be detected by this method, indicating a decrease in amplification efficiency that either due to skewing effect or a negative impact of human genomic DNA on amplifying low amount of HPV68 DNA.

**Figure 3:**
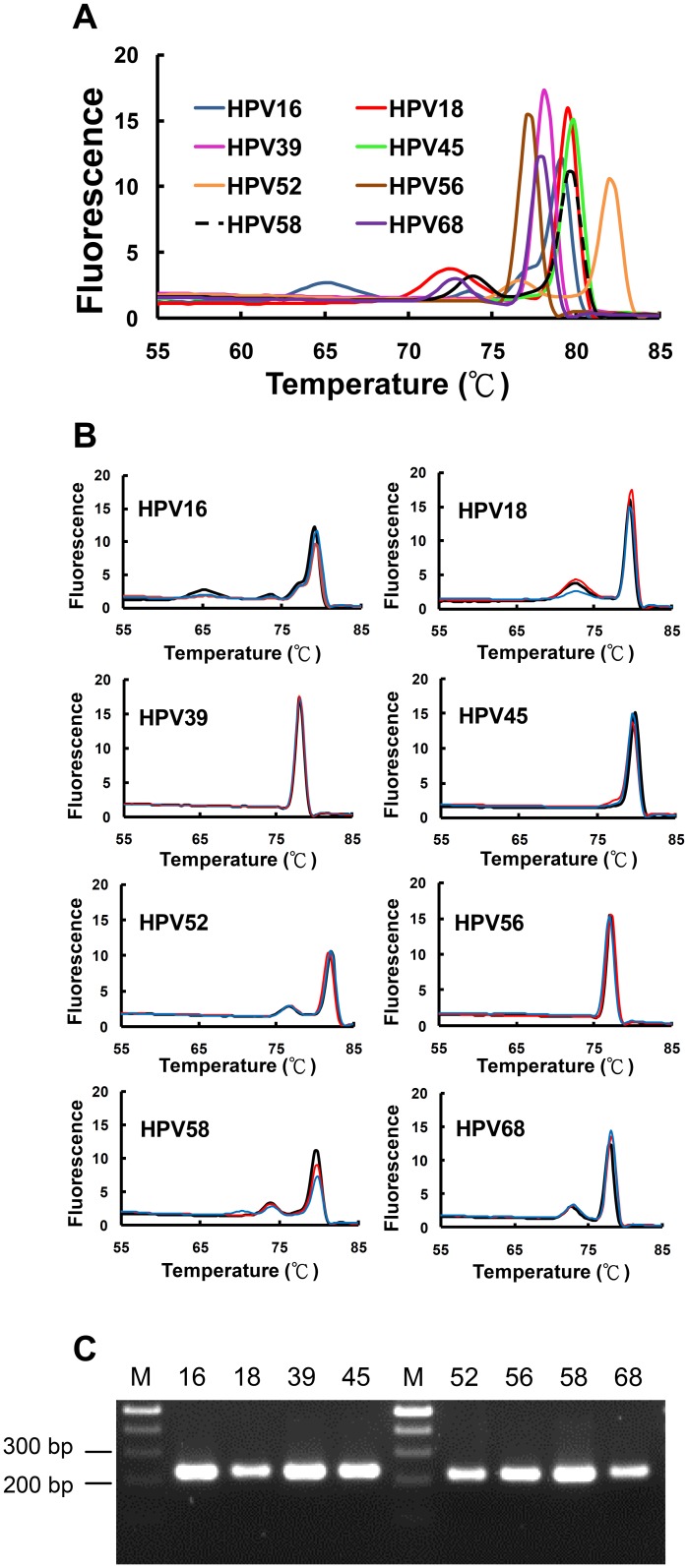
High-resolution derivative plots for 8 common and high-risk HPV genotypes. A. The plasmids carrying genotype-specific DNA fragment were subject to asymmetric broad-range real-time PCR followed by HRM analysis. The derivative plots for all 8 genotypes were plotted together to reveal the differences among different HPV genotypes. B. Two clinical samples (red and blue curves) for each indicated genotype were subject to asymmetric broad-range real-time PCR and HRM analysis. Derivative plots for the indicated HPV genotypes were plotted together with the derivative plot obtained from plasmid DNA (black curve) to reveal the consistent melting patterns between these two different sources of template DNA. (C) Agarose gel electrophoresis was performed to demonstrate the generation of genotype-specific PCR products from asymmetric broad-range real-time PCR.

The HRM profiles for 8 high-risk HPV genotypes were then generated by asymmetric broad-range real-time PCR in the presence of the unlabeled probes specific for HPV16 and HPV18. The melting temperatures and melting profiles provide molecular signatures for the 8 high-risk genotypes that can be unambiguously identified through high-resolution derivative plots (Table 2 and Fig. 3A). When clinical samples with the indicated genotypes were subject to the analyses, the melting patterns were in accord with those obtained from plasmid DNA template (Fig. 3B). Agarose gel electrophoresis further confirmed the generation of genotype-specific PCR products (Fig. 3C). The distinct HRM molecular signatures thereby provide a basis for genotyping of HPV subtypes.

**Table 2:** High-resolution melting profiles for high risk HPV genotypes disclosed by broad-range real-time PCR.

HPV genotype (n)^a^	T_m_ ± SD^b^ (°C)	GC content (%)	GenBank accession no.
HPV16 (5)	65.09±0.4979.10±0.17	34	NC001526.1
HPV18 (5)	72.45±0.2679.53±0.27	36	AY262282.1
HPV39 (5)	78.26±0.20	36	M62849.1
HPV45 (5)	79.81±0.17	36	X74479.1
HPV52 (5)	76.70±0.2782.22±0.21	36	EU077211.1
HPV56 (4)	77.07±0.03	33	EF177176.1
HPV58 (5)	73.87±0.2879.74±0.21	34	D90400.1
HPV68 (5)	72.96±0.2278.12±0.03	36	DQ080079.1

a Number of test (strain).

b The variance between 4–6 measurements with the indicated number of isolates.

We further assessed our method in HPV genotyping of clinical specimens retrospectively. A total of 140 clinical samples from patients who were suspected of having precancerous lesion were blind tested. Due to the nature of sample storage, only 119 of the 140 DNA samples were informative and generated PCR products that were suited for HRM analysis (Table 3). As revealed by EasyChip genotyping and DNA sequencing, 70 of the 119 informative cases were infected with high-risk HPV subtypes, while 49 were infected with low-risk subtypes. In addition, 65 of the 70 samples were infected with the HPV subtypes that were the analytical subjects of this study. Of the 65 samples, 59 displayed distinguishable HRM patterns that can be assigned to the correct genotype with the typing rate equivalent to 91%. According to EasyChip genotyping analysis, the remaining 6 samples were infected with multiple HPV genotypes and can not be genotyped accurately by HRM analysis (Table S1).

**Table 3:** The validation assay for 119 clinical HPV isolates.

HPV type	Genotype	No. of isolates tested	No. of isolates assigned with correct genotype	Correct genotyping rate (%)
High-risk	HPV16	15	15	100
	HPV18	2	2	100
	HPV39	11	10	91
	HPV45	5	5	100
	HPV52	8	7	88
	HPV56	3	2	67
	HPV58	14	12	86
	HPV68	7	6	86
	Total	65	59	91
	Others^a^	5	-	-
Low-risk	HPV42	7	6	86
	HPV62	7	7	100
	HPV70	6	5	83
	CP8304	6	6	100
	CP8061	2	2	100
	MM8	4	3	75
	Total	32	29	91
	Others^b^	17	-	-

a High-risk HPV genotypes that are not characterized in this study.

b Low-risk HPV genotypes that are not characterized in this study.

**Figure 4:**
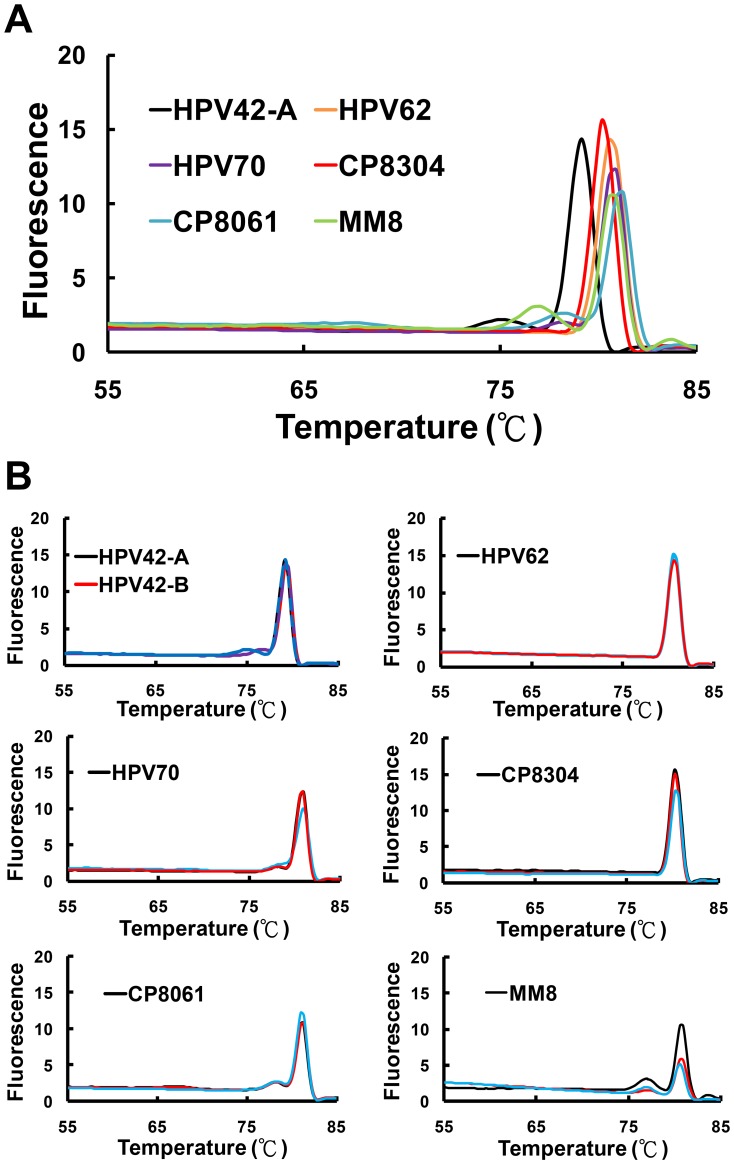
Unique high-resolution derivative plots for 6 low-risk HPV genotypes. Clinical isolates that did not have derivative plots typical of those in our HRM database were analyzed. The HPV42, 62, 70, CP8304, CP8061 and MM8 genotypes were found to display their unique high-resolution derivative plots. The derivative plots were plotted together to reveal the differences among different HPV genotypes (panel A). The derivative plots for two to three measurements of each HPV genotypes were plotted to reveal the minimal inter-assay variability (panel B). Note the consistent derivative plot pattern for each virus subtype.

During our analysis of clinical specimens, several low-risk HPV genotypes including HPV42, 62, 70, CP8304, CP8061 and MM8 were found to display their unique high-resolution derivative plots (Fig. 4A and 4B). Of the 49 specimens that were infected with low-risk genotypes, 32 of them belonged to HPV42, 62, 70, CP8304, CP8061 and MM8 with 29 of them being genotyped accurately. The correct genotyping rate reached 91% (Table 3). Furthermore, 2 cases of HPV11 and 2 cases of HPV81 that were assigned as “others” (Table 3) in the low-risk group were, at the beginning, mistakenly classified as HPV54 and CP8304, respectively. All the rest of the samples (n = 18) assigned in the “others” group for both high-risk and low-risk genotypes was not falsely claimed as infected with the HPV subtypes of interest. Together, an HRM database for a total of 14 HPV genotypes was established that form the basis for HPV genotyping.

## Discussion

HPV is well recognized as a major cause of cervical cancer. The development of high grade pre-cancer and invasive cervical cancer could be due to persistent high-risk HPV infection. A rapid, accurate and sensitive method to detect and differentiate HPV genotypes is essential to identify high risk patients who are otherwise found to have normal cytological results and women with cervical cancer potential from the screened population. HPV genotyping provides a reference point for HPV vaccination and HPV prevalence in natural history studies during clinical practice. Unlike hepatitis B vaccine, there is no acceptable antibody test for HPV vaccines. If it is affordable, HPV genotyping assay should be the first choice for HPV testing. In this study, a novel approach for HPV genotyping is developed. This method is based on the combined use of asymmetric broad-range real-time PCR and HRM analysis. Accordingly, 8 high-risk and 6 low-risk HPV subtypes can be identified that offers a novel approach for HPV genotyping.

A number of HPV DNA genotyping methods including real-time multiplex PCR, Hybrid Capture II INNO-LiPA v2 HPV genotyping PCR, Roche Amplicor MWP HPV test and Digene HC2 assay have been reported in the literature [16], [41]–[45]. Recent trends in the application of HRM analysis for microorganism identification [30], [31], [35] lead us to explore a new avenue to identify and differentiate HPV genotypes. Through bioinformatic analysis, we revealed that the 153–163 bp interprimer regions of FRG5 and FRG2 is relatively conserved within isolates of the same genotype, whereas nucleotide divergence is present among different HPV genotypes. Therefore, the PCR amplicon likely contains information for at least partial phylogenetic characterization for HPV genotyping. We demonstrated that 8 high-risk and 6 low-risk HPV genotypes can be identified through the HRM molecular signatures that were generated by asymmetric broad-range real-time PCR of the L1 DNA fragment in the presence of two HPV16- and HPV18-specific unlabeled probes. The relatively small PCR amplicon also results in an increase in the detection limit of this method when compared with the previously reported broad-range PCR such as MY09/11 that has a drawback of a large PCR fragment with less sensitivity [21]. Our data indicate that as little as 10 ag of plasmid DNA carrying the HPV18 PCR amplicon equivalent to 3 copies of HPV genome is detectable by this method. Due to the 2 mismatches of the last 7 nucleotides in the FRG5 primer region, the detection limit for HPV68 is slightly affected with 30 copies of genome being detected. As judged by the Ct value obtained from the analysis of clinical specimens, most of the samples contained more than 300 copies of HPV DNA. Hence, this assay should have sufficient sensitivity for most of the clinical HPV genotyping analysis. When it is combined with rapid-cycle PCR, HRM analysis requires minimal time, and the material cost is usually less than $3. The time required for the differentiation of HPV genotypes is considerably short when PCR is performed directly with clinical specimens. Approximate 3 h is required for genotyping of the clinical samples with the isolated DNA as the starting material.

Multi-infection of HPV strains with various genotypes accounts for approximate 8–22% of the HPV-infected patients [46]–[48]. The prevalence of multiple HPV infections varies in relation to the method used to detect HPV DNA and the study population. In addition, a potential skewing for amplification of HPV DNA in specimens containing multiple HPV genotypes by PCR with broad-range primers has been reported [49]. A technical limitation of the current method is the incapability to clearly identify the genotypes in these scenarios. Hence, among the clinical samples we analyzed in this study, the HPV genotypes for 7 HPV-positive samples that are infected with multiple HPV genotypes can not be identified by HRM analysis. However, the distinguished HRM patterns provide a hint that multiple infections may occur that required further identification with other methods.

In conclusion, the molecular signatures from HRM analysis of the broad-range real-time PCR products are useful for detection and genotyping of HPV infection. This approach can be extended further to cover all the 13 high-risk HPV genotypes. With an appropriate HRM molecular signatures database, this method should allow rapid and cost-effective differentiation of HPV genotypes.

## Supporting Information

Figure S1 **Effects of human genomic DNA on the amplification of HPV DNA.** A–C. Serial dilution of the plasmid harboring the L1 fragment of HPV18 (panel A and C) or HPV68 (panel B and C) corresponding to the indicated copy number of template DNA were subject to amplification by asymmetric broad-range real-time PCR in the presence or absence of 20 ng of human genomic DNA. The copy numbers of the template DNA were plotted against Ct values.
(DOC)

Table S1 **The genotypes for the samples with multiple infection.**
(DOC)
