# Supplementary material for: High-Resolution Melting Molecular Signatures for Rapid Identification of Human Papillomavirus Genotypes
Source: PLoS One. 2012 Aug 20;7(8):e42051. doi: 10.1371/journal.pone.0042051 (PMC3423390; doi:10.1371/journal.pone.0042051)
Supplement: Figure S1 — Effects of human genomic DNA on the amplification of HPV DNA. A–C. Serial dilution of the plasmid harboring the L1 fragment of HPV18 (panel A and C) or HPV68 (panel B and C) corresponding to the indicated copy number of template DNA were subject to amplification by asymmetric broad-range real-time PCR in the presence or absence of 20 ng of human genomic DNA. The copy numbers of the template DNA were plotted against Ct values. (DOC) [file pone.0042051.s001.doc]

**Figure** **S1** Effects of human genomic DNA on the amplification of HPV DNA. A-C. Serial dilution of the plasmid harboring the L1 fragment of HPV18 (panel A and C) or HPV68 (panel B and C) corresponding to the indicated copy number of template DNA were subject to amplification by asymmetric broad-range real-time PCR in the presence or absence of 20 ng of human genomic DNA. The copy numbers of the template DNA were plotted against Ct values.
