# Supplementary material for: High-Resolution Melting Molecular Signatures for Rapid Identification of Human Papillomavirus Genotypes
Source: PLoS One. 2012 Aug 20;7(8):e42051. doi: 10.1371/journal.pone.0042051 (PMC3423390; doi:10.1371/journal.pone.0042051)
Supplement: Table S1 — The genotypes for the samples with multiple infection. (DOC) [file pone.0042051.s002.doc]

**Table S1. The genotypes for**

**the samples with multiple infection**

| **Case Number** | **Genotype by EasyChip** |
| --- | --- |
| 1 | 39, 58, MM8 |
| 2 | 52, 67, MM8 |
| 3 | 56, CP8304 |
| 4 | 54, 58 |
| 5 | 58, 70 |
| 6 | 58, 68 |
